# White Coat Hypertension & Cardiovascular Outcomes

**DOI:** 10.1007/s11906-024-01309-0

**Published:** 2024-05-18

**Authors:** Raymond R. Townsend, Jordana B. Cohen

**Affiliations:** Perelman School of Medicine at the University of Pennsylvania, Renal Electrolyte and Hypertension Division, 122 Founders Building 3400 Spruce Street, Philadelphia, PA United States

**Keywords:** White coat effect, White coat hypertension, White coat uncontrolled hypertension, Cardiovascular risk

## Abstract

**Purpose of Review:**

This review aims to inform the reader of the complexity of blood pressure responses when comparing blood pressure measured in the medical environment to that outside the medical environment. In addition, we summarize what is known about current predictors of white coat hypertension, reevaluate the relationship of white coat hypertension to cardiovascular outcomes, and provide some clinical guidance on management.

**Recent Findings:**

Differences in outcomes exist when white coat effect occurs in unmedicated people versus the white coat effects in those on antihypertensive therapy.

**Summary:**

White coat hypertension is relatively common, carries a small but definite increase in cardiovascular risk, and is prone to conversion to sustained hypertension. Future research will hopefully tease out the roles of ancillary findings that characterize a white coat hypertensive (like modest elevations in creatinine, glucose and triglycerides) in the elevated cardiovascular risk, and test the effectiveness of mitigation strategies in these patients.

## Introduction

At present, our presupposition is that most readers will have an idea about what a “white coat effect” is. Pursuing that further, ask yourself the following seven questions:

- How would you define a white coat effect?
- Does it matter how you measure blood pressure when defining a white coat effect?
- Are you familiar with the “alerting reaction” and why it may be important here?
- When white coat effects are present in an individual, does it matter if they are taking medications for high blood pressure?
- When white coat effects are present in an individual, does it matter if they have clinical, or even subclinical, organ damage typically associated with high blood pressure?
- When you identify a white coat effect in a patient, is it reproducible?
- When you identify a white coat effect in a patient and what do you do about it?
- If you already know the answers to these questions, you probably won’t learn much from this review. If, like the authors, you find yourself intrigued by these questions, please—read on!

### History and Definitions

As clinical care providers we generally assume that office blood pressures (OBPs) can reflect a transient elevation of BP induced by the medical environment but are not necessarily representative of the patient’s usual daytime BPs when we encounter in-office BP higher than the currently accepted goal BP. A moment’s pause will show, however, that even when a patient has a normal OBP, for example 114/78 mmHg, carefully measured home values may average 102/68 mmHg. Is that white coat hypertension, or white coat effect, or a white coat phenomenon? Thus, like ‘hypertension’, there is a devil in the definitional details, and a need to communicate clearly about this topic.

#### Definition(s) of White Coat Terms

- It is important at the outset to define four terms used in this report:
  - White Coat Phenomenon (WCP).
  - White Coat Effect (WCE).
  - White Coat Hypertension (WCH) and.
  - White Coat UnControlled Hypertension (WUCH).

The WCP refers to the difference between a BP measured in the office and a BP measured outside the office setting, wherein the OBP is higher than the out of office one. It does not depend on a threshold value, thus applies to patients with and without ‘hypertension’, and is a label used to underscore the commonly found differences between in-office versus out-of-office BP values when they are discordant, **and** the in-office values are higher.

WCP is the phenomenon where the BP in an office setting is higher than current guidelines recommend (thus in the ‘hypertensive’ range), whether or not a person is on BP medication, while the out-of-office BP (in the same person) is below current guideline recommendation for out-of-office BP goals. The out-of-office values are often measured with daytime ambulatory BP, but could also be undertaken using well done home BP measurements.

The White Coat Effect (WCE) is the magnitude of the difference between the in-office and out-of-office values. WCEs are usually reported as both the difference in SBP (often with a measure of variation) and DBP (also often with a measure of variation).

As an example: consider a 44 year old man whose OBP is measured correctly and is reported as 136/77 mmHg, representing an average of the two office visits. He is untreated with BP medications. The health care provider orders a 24-h ABPM and his daytime BPs (an average of 28 readings between 7 AM and 9 PM) are 123(± 12) / 73 (± 8) mmHg. He exhibits a WCP in that there is a substantial difference between the in-office and the ambulatory values. He has WCH since his in-office values are higher than the currently recommended threshold value of 130 mmHg systolic [1], and his daytime ambulatory systolic pressure is lower than the current recommended ambulatory daytime threshold value of 130 mmHg in the absence of antihypertensive medication [2]. The magnitude of the WCE in this patient is 13/4 mmHg.

Since studies of white coat observations have included patients taking and not-taking antihypertensive medication, WCH generally refers to those **not** on medications, and WUCH refers to those **on antihypertensive medication** who remain uncontrolled in the in-office setting. Our example falls into the category of WCH. If he were taking an antihypertensive medication, and had the same findings, he would be labelled as WUCH.

#### Alerting Reaction

The alerting reaction describes the relationship between properly taken serial BP measurements, undertaken over several minutes, where the first and sometimes the second values are different, and usually higher, than subsequent values [3]. It can occur in the office setting, or outside the office. An example is shown below:

- 09:28 AM 131/75 mmHg; 76 beats/minute.
- 09:30 AM 126/74 mmHg; 73 beats/minute.
- 09:32 AM 125/74 mmHg; 73 beats/minute.

In a pair of studies published in the 1980s, Mancia and colleagues investigated the effects of a physician or a nurse entering a patient’s room on their BP, including the ‘alerting’ effects of inflating the cuff itself [4, 5]. In both studies, patients were in bed with an established intra-arterial continuous pressure monitor in the radial artery during a planned visit by a physician who was unknown to the patient. Summarizing these two studies, done in 94 patients, including normotensive and untreated hypertensive patients, they observed:

- A rise in systolic (by 17–27 mmHg) and diastolic (by 15–19 mmHg) BPs as soon as the physician entered the room, which peaked by 4 min and subsided, but not quite to baseline, after the 10–15 min visit by the physician was over
- Heart rate increased an average of 13–18 beats/minute in the same timeframe
- Most patients had the same BP and heart rate response when the visit was repeated
- A rise in BP happened in almost everyone, and was not dependent on age, patient sex, or starting BP; inflating a standard BP cuff on the non-catheterized arm did not influence the BP further
- When a comparison was made between a male physician and female nurse, the rise in BP and heart rate still occurred with the nurse visit, but was about 40–50% of the values observed during the physician-conducted the visit
- The manual cuff inflations, in both studies, were not associated with changes in intra-arterial BP recordings, or changes in heart rate

The mechanism of the alerting reaction is not known, but strongly implies a sympathetic activation triggered by the health care worker entering the room.

##### Summarizing Definitions

The measurement of BP produces a number of interesting, though not necessarily interdependent phenomena. The discrepancy where OBPs are higher than those outside the office, termed WCP, happens to about 20% of unmedicated people who are normotensive when checked outside the office, and this figure is likely higher (in the range of 40% or more) for those on antihypertensive medication who appear to have controlled BP outside the office [6]. The alerting reaction happens to about 3 out of 4 people, but is only poorly correlated to WCP. The effect of a physician or a nurse on BP and heart rate is separate from the effects of cuff inflation per se, but in both instances there is a peak effect and typically a decline in subsequent readings over time.

### Mechanisms and Associations WCE

There are limited numbers of investigations into mechanisms of WCP, so we are concentrating on associations, and provide mechanistic inferences when plausible.

In an early study of over 2000 unmedicated participants with OBP ≥ 140/90 mmHg, free of hypertension mediated organ damage, who lacked evidence of secondary hypertension and underwent ABPM assessments (with daytime BP ≤ 135/85 mmHg defining WCH) the investigators observed the following [7]:

- Older age was more likely to show WCEs.
- Women were more likely than men to have WCEs.
- Smokers were less likely to show WCEs.

These results make sense when you consider that older age tends to be associated with an elevation in SBP and greater BP variability. Greater BP variability likely is present in daily life circumstances that impose a stress on the autonomic nervous system where the response is potentially exaggerated due to arterial stiffness, reduced baroreceptor function or heightened autonomic responsiveness [8••]. The autonomic system, in particular, is likely an important contributor to what happens to OBP values, particularly when BP measurements are attended by a doctor [9]. Cigarette smoking exposes one to nicotine, which has a BP elevating effect. Consistent with the findings in this study, other investigations have shown that for the same level of OBP, smokers have higher ABPM values compared to non-smokers, and would be less likely to have WCEs [10].

A study in patients with OBP of more than 140/90 mmHg and daytime ABPM less than 135/85 mmHg was conducted in those on, and those not on, antihypertensive medications [11•]. The two cohorts were combined for this study, yielding a total of 1434 participants. They used a machine learning algorithm to evaluate up to 26 potentially informative, patient-related variables, to predict likelihood of WCE. They derived several prediction models and settled on the Random Forest matrix plot as the best approach from the standpoint of the achieving highest area under the receiver operator characteristics curve and average precision values. They found that the five most predictive variables, rank ordered, were office diastolic pressure, followed by office systolic pressure, kidney function (estimated glomerular filtration rate), blood glucose concentration and current smoking. Higher office DBP and SBP values were more likely to have WCE. Lower eGFR was more likely to have WCE. Higher glucose concentrations were more predictive of classifying a participant into the WC group. The tendency of higher glucose and higher triglyceride levels, diagnostic elements in the metabolic syndrome, were also noted to be higher in participants with WCH defined as office values ≥ 140/90 mmHg and home BP values ≤ 135/85 mmHg [12].

A study from China used routinely available clinical data (including 17 items, like age, gender, SBP, DBP, BMI, and labs), excluding participants taking antihypertensive medications or with a serum creatinine ≥ 133 umol/L (1.5 mg/dL), obtained on 553 people to determine if a model could be constructed that predicted WCH from an office visit with elevated OBPs [13]. They used a Least Absolute Shrinkage and Selection Operator (LASSO) approach, which is a form of regression that attempts to avoid overfitting variables. LASSO performs both variable selection, and uses a process called regularization to enhance accuracy in predicting and interpreting the model. They divided their cohort into a training set (n = 445) and validation sets (n = 108 in each set). ABPM confirmed whether OBP elevations were sustained, or represented WCEs. Participants were enrolled when OBP was ≥ 140/90 mmHg (done in triplicate), and the definition of sustained hypertension by ABPM was any of the following: 24 h ABP ≥ 130/80 mmHg; daytime ABPM ≥ 135/85 mmHg; nighttime BP ≥ 120/70 mmHg. They observed that six variables were found useful by the LASSO regression method in predicting WCH, and these included isolated systolic hypertension (ISH), the SBP, the DBP, the serum triglyceride concentration, the serum creatinine, and a positive history for cardio- and cerebrovascular disorders. Finding that in-office ISH predicts WCH in this study supports prior investigations that have also noted association between ISH and IDH with both WCH and WUCH [14]. In the Chinese machine learning study, higher levels of triglyceride and creatinine were more prevalent in WCH compared with normotensive in- and out-of-office, a finding again supported by other studies such as such as Pressioni Arteriose Monitorate e Loro Associazioni (PAMELA) study, which brought focus to the dysmetabolic environment in WCH, which may explain why some studies show an increase in cardiovascular risk when WCH is present [8••].

#### Summarizing Associations (**see **Fig. 1)

A suspicious profile for a WCE could include a patient whose office readings, done well, are elevated and who has:

- OBPs that are either very close to cut off range for hypertension, or selective elevations are present in that systolic is high and diastolic is not (ISH), or vice versa (IDH).
- Poorer kidney function.
- Non-smoker.
- Women (in some studies).
- Older adults.
- A tendency to higher levels of metabolic risk factors such as serum triglycerides and glucose concentrations.

**Fig. 1:**
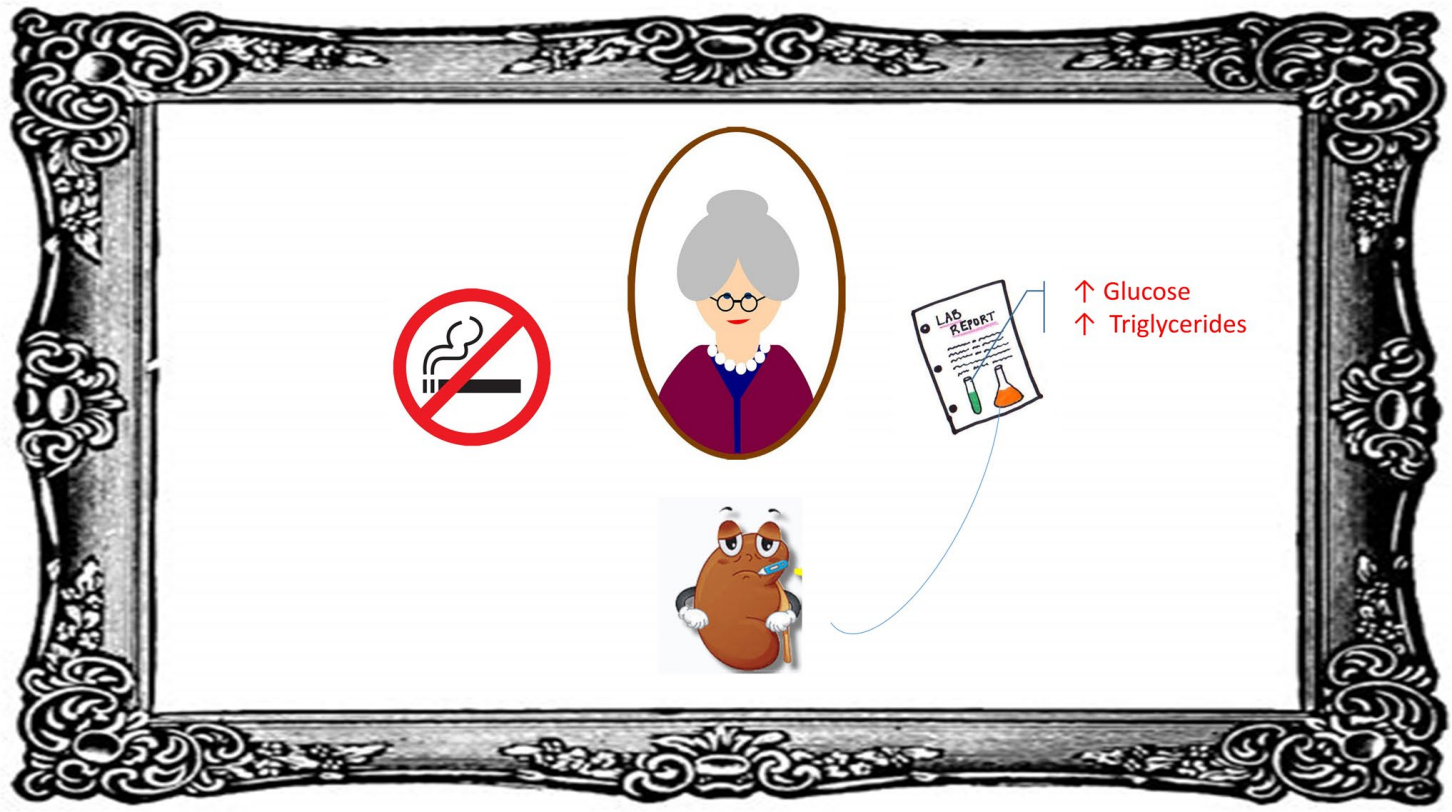
Graphic portrayal of factors or findings often associated with white coat hypertension. Pictured are the elderly, particularly women, laboratory findings that associate with higher cardiovascular risk (glucose and triglycerides), reduced kidney function and non-smoking status. See text for details

## Prognosis of WCH and WUCH

Whether WCH and WUCH portend negative long-term outcomes has long been a point of debate. The uncertainty surrounding the prognosis of WCH and WUCH is largely driven by inconsistent findings across studies. Most studies have identified no notable association of WCE with risk of cardiovascular events and/or mortality [15–33]. However, several studies have identified slightly and even markedly elevated risk from WCE [34–37], raising concerns that the risks may be understated.

Notably, in 2018, Banegas et al. evaluated 63,910 participants of the Spanish Ambulatory BP Registry followed for a median of 5 years and identified a markedly increased risk of cardiovascular death (hazard ratio [HR] 1.96, 95% confidence interval [CI] 1.22–3.15) and all-cause death (HR 1.79, 95% CI 1.38–2.32) associated with WCH (35). This study was retracted two years after publication [38]. A more recent publication evaluating 59,124 participants of the Spanish Ambulatory BP Registry, followed for a median of 10 years, found no increased risk of cardiovascular or all-cause death from WCH [19•].

Given the inconsistencies in the prognostic role of WCE across studies, multiple systematic reviews and meta-analyses have aimed to clarify the cardiovascular and mortality risks of WCE (**see **Table 1) [39–42, 39, 40]. However, interpretation of these meta-analyses was limited due to a small number of available studies at the time, resulting in some suboptimal analytic approaches [43] and hampering stratification by antihypertensive treatment status and other potential sources of heterogeneity.

**Table 1:** Meta-Analysis Results Evaluating the Relationship of WCH and WUCH with Risk of Cardiovascular Events and All-Cause Mortality

Meta-analysis	Number of studies (participants)	Cardiovascular Events, HR (95% CI)			All-Cause Mortality, HR (95% CI)		
		Mixed WCH + WUCH	WCH	WUCH	Mixed WCH + WUCH	WCH	WUCH
Fagard and Cornilissen [39]	7 (11,502)	1.12 (0.84–1.50)					
Briasoulis et al. [40]	14 (29,100)	1.73 (1.27–2.36)			1.50 (0.86–2.62)		
Huang et al. [41]	14 (21,336)	1.19 (1.01–1.41)	1.38 (1.15–1.65)	1.16 (0.91–1.49)		1.20 (1.03–1.40)	
Cohen et al. [42••]	27 (64,273)	1.26 (0.95–1.73)	1.36 (1.03–2.00)	1.12 (0.91–1.39)	1.43 (1.13–1.82)	1.33 (1.07–1.67)	1.11 (0.89–1.46)

In a more recent meta-analysis, we analyzed 27 studies that evaluated the cardiovascular and mortality risk of WCH and WUCH [42••]. We identified that WUCH was not associated with any increased risk, but that WCH was associated with a modestly increased risk of both cardiovascular events (pooled HR 1.36, 95% CI 1.03–2.00) and all-cause death (pooled HR 1.33, 95% CI 1.07–1.67), which persisted in sensitivity analyses excluding the Banegas study (cardiovascular events pooled HR 1.29, 95% CI 1.06–1.61). Among studies that did not stratify by antihypertensive treatment status but in which less than half of participants were prescribed antihypertensive medications, we similarly observed an increased risk of cardiovascular events and all-cause death among participants with WCE [16, 24–26, 36, 37]. When at least half of participants were prescribed antihypertensive medications, we found no association of WCE with cardiovascular events or all-cause death [27–30, 33]. Thus, we postulated that combining data from treated and untreated participants may have contributed to some of the mixed findings in prior studies.

In sensitivity analyses, we found that the magnitude of the associations of WCH with adverse outcomes were strongest among studies that included older participants and those with previous cardiovascular and kidney disease, which corroborated prior observations by Franklin et al. in the International Database of Ambulatory BP in Relation to Cardiovascular Outcomes [22]. We also observed that WCH did not seem to be associated with increased risk of stroke [30, 40–46]. Additionally, compared to normotensive participants, those with WCH had a 3- to fourfold higher risk of developing sustained hypertension over seven to ten years of follow-up [26, 27]. Furthermore, we found that the cardiovascular risk of WCH was strongest among studies with ≥ 5 years of follow-up time. We hypothesized that longer duration of follow-up may capture a stronger association of WCH with adverse outcomes due to more cases of WCH having time to convert to sustained hypertension during the longer timeframe. An accompanying editorial elegantly emphasized that the magnitude of risk from WCH in our meta-analysis was much lower than that of sustained hypertension, upon meta-analyzing data from the same studies (cardiovascular events pooled HR 2.31, 95% CI 1.91–3.15; all-cause death pooled HR 1.44, 95% CI 1.25–1.66) [47].

### Summarizing Prognosis

The cardiovascular and mortality risk of WCE is inconsistent across studies. Meta-analytic data suggest that WUCH is not associated with any elevated risk. WCH may be modestly associated with elevated risk of cardiovascular events and all-cause mortality, but this risk is markedly lower in magnitude than that of sustained hypertension. The cardiovascular risk from WCH seems potentially driven by a strikingly elevated risk of transitioning to sustained hypertension.

## Are WCE Reproducible?

There is far more data published on the prevalence of WCE than there is on its reproducibility. The literature that does exist shows several interesting points.

The reproducibility of the WCE in initially untreated patients was evaluated in the European Lacidipine Study of Atherosclerosis (ELSA) [48]. In this investigation, the investigators recruited more than 1600 participants, and in-office and ABPM studies were compared at Baseline and then at yearly follow up (after blinded treatment was started: participants were randomized to either atenolol or lacidipine) for the next 4 years. At 1 year follow up, the persistence of a WCE in those who had it at Baseline (prior to receiving randomized blinded drug therapy) was 40%. By the fourth year, whether a participant was randomized to the atenolol arm, or to the lacidipine arm, WUCH persisted in < 5%.

In the recent study of Palatini and colleagues, they examined the robustness of a diagnosis of WCH (untreated, found by elevated office values of > 140/90 mmHg) using data from the Hypertension and Ambulatory Recording Venetia Study (HARVEST), an observational cohort of 1050 people who were 18–45 year old at low cardiovascular risk, with in-office stage 1 hypertension who were unmedicated [49]. HARVEST began enrolling in Northeast Italy 1990, when European Guidelines for initiating antihypertensive therapy stipulated OBP of ≥ 160/100 mmHg. The primary objective of HARVEST was to monitor how often these low risk patients exceeded the threshold to begin antihypertensive drug therapy. The in-office and ABPM were repeated three months after enrollment, at which time only one third of the 1050 participants continued to have WCH.

In one of the early WCE reproducibility studies of a mixed population of treated, and untreated with antihypertensive medication referred (for whatever reason) to have an ABPM, Ben-Dov and colleagues evaluated persistence of WCE in 196 patients who had their ABPM repeated, on average one and one-half years later [50]. They defined WCE as OBP ≥ 140/90 mmHg and daytime ABPM < 135/85 mmHg. Of 31 patients noted to have WCE on their first ABPM, 19 of these (61%) persisted in having a WCE on the second reading.

Finally, an interesting observation from the Spanish ABPM registry is of note here [51•]. In an investigation of 839 untreated patients who had in-office hypertension (> 140/90 mmHg) preceding the first of several repeated ABPM measurements the investigators noted that if the office and ABPM measures were repeated within a month, as was done in 325 participants, 89% continued to show WCH with an ABPM that continued to reflect a 24 h BP < 130/80 mmHg). In the remaining 514 participants, where the median time between ABPM measurements was 11 months, WCH persisted in 42%, not unlike the studies covered above.

### Summarizing Reproducibility

In untreated WCH, reproducibility is relatively high if done again within a month, but falls dramatically after 3 months. In treated WUCH, comparing baseline prevalence prior to drug treatment with persistence of WCE over time, the persistence of WUCH falls dramatically with each passing year, to < 5% at four years.

Patients with WCEs, and patients with other effects like masked hypertension, controlled in-office and out of office, and uncontrolled in both environments show a remarkable switching to a different form of in-office versus out of office category. Moral of story: you won’t know unless you are checking. At this time, yearly recheck in the patient with untreated WCH is reasonable, and for the WUCH patients on treatment it will depend on the demographics and co-morbidities and requires clinical judgment to determine an optimal follow up strategy.

## Treating WCE

Patients with WCH and WUCH have the potential to be harmed by initiation or escalation of antihypertensive therapy based on OBP readings alone. These individuals may be subject to hypotensive effects of unnecessary therapy, which in extreme cases could realistically result in traumatic falls and ischemic events (e.g., similar to that observed in treatment of inpatient elevations in BP [52, 53]). Additionally, patients with WCH and WUCH are susceptible to experiencing BP-independent adverse effects from treatment with unnecessary medications.

There is a dearth of data to guide cardiovascular risk attenuation in WCH. However, the potential cardiovascular risk from WCH may be safely mitigated by lifestyle modifications (1) and close monitoring for transition to sustained hypertension [26, 37]. To achieve the latter, we typically recommend that these patients undergo ABPM annually (or biannually if they are at low cardiovascular risk), and self-monitor their BPs at home using a validated BP device [54] for a minimum of three days monthly, performing two readings in the morning and two readings in the evening with appropriate technique [55].

## Future Directions

Greater use of in-office automated BP measurements with devices that can take multiple measurements of BP without the presence of a health care provider in the office may reduce the prevalence of WCH and lessen the burden of multiple home measurements and ABPM [56].

## Conclusions

As authors, it is our sincere intent to function more as a sail, than as an anchor, in the navigation of WCE. The in-office environment, and the out-of-office environments are clearly dissimilar, so it is understandable that BP values will vary from the office, whether taken well at home, or by using an ambulatory monitor. What likely makes a WCE more clinically impactful is an appreciation of the magnitude of the differences in the BP in the two locales, the timing of a retest in the out-of-office environment, the usage of antihypertensive medication, and the presence of key demographics and co-morbidities as outlined above. The main thing is that when found, particularly in an untreated patient, it is important to not dismiss it as clinically harmless since follow up studies show a substantial relocation from WCH to sustained hypertension, along with evidence of progressive hypertension mediated organ effects emerging, like left ventricular hypertrophy.

## Data Availability

Not applicable.

## Abbreviations

ABPM: Ambulatory Blood Pressure Monitor
BP: Blood Pressure
DBP: Diastolic Blood Pressure
IDH: Isolated diastolic hypertension
ISH: Isolated Systolic Hypertension
mmHg: millimeters of mercury
OBP: Office Blood Pressure
SBP: Systolic Blood Pressure
WC***x***: placeholder term for white coat possibilities including where ‘x’ = “**E**ffect [WCE]”, “**H**ypertension [WCH]”, “**U**n**C**ontrolled **H**ypertension [WUCH]” and “**P**henomenon [WCP]”)
